# Sensory Processing Sensitivity and Maladaptive Personality Traits in Chronic Pain Conditions: A Network Analysis Perspective

**DOI:** 10.1155/prm/8005108

**Published:** 2026-03-28

**Authors:** Marco Cavicchioli, Filippo Maria Nimbi, Sara Bottiroli, Daniele Guglielmi, Lorys Castelli, Federica Galli

**Affiliations:** ^1^ Department of Dynamic and Clinical Psychology, and Health Studies, Faculty of Medicine and Psychology, SAPIENZA University of Rome, Rome, Italy, uniroma1.it; ^2^ Department of Psychology, Sigmund Freud University, Milan, Italy, milano-sfu.it; ^3^ Department of Brain and Behavioral Sciences, University of Pavia, Pavia, Italy, unipv.eu; ^4^ IRCCS Mondino Foundation, Pavia, Italy, mondino.it; ^5^ Department of Psychology, University of Turin, Turin, Italy, unito.it

**Keywords:** altered emotion processing, chronic pain, maladaptive personality traits, self-other dysfunctions, sensory processing sensitivity

## Abstract

**Background:**

There is a growing interest in clarifying factors involved in the emergence of different chronic pain (CP) conditions, especially referring to the role of personality factors. Accordingly, the current study aims at exploring implications of temperamental (sensory processing sensitivity) and maladaptive personality traits across different CP syndromes (i.e., chronic headache, vulvodynia, and fibromyalgia) in patients with comorbid conditions compared to healthy controls (HCs).

**Methods:**

The sample included 1144 women (chronic headache: 222; vulvodynia: 221; fibromyalgia: 201; comorbid CP conditions: 359; HC: 141). Participants completed an online self‐report survey composed of the Highly Sensitive Person Scale (i.e., Aesthetic Sensitivity, Low Sensory Threshold [LST], and Ease of Arousal [EOE]) and the Personality Inventory for the DSM‐5 Short Form (Negative Affectivity [NA], Detachment [DE], Antagonism, Disinhibition, and Psychoticism [PSY]). MANCOVA and distinct network analysis for each group were conducted.

**Results:**

CP conditions were characterized by higher levels of LST and EOE together with NA, DE, and PSY than HCs. NA, DE, and PSY were the central nodes of the personality traits network in CP conditions. DE and PSY were the most representative traits among patients with comorbid CP syndromes.

**Conclusions:**

Temperamental and maladaptive personality traits reflecting altered affective processing systems should be considered common factors involved in different CP syndromes. Dissociative/self‐related processes and dysfunctions in interpersonal functioning should be systematically evaluated as possible core markers of a clinical group composed of patients with comorbid CP conditions, as well as relevant targets for psychotherapeutic interventions.

## 1. Introduction

Chronic pain (CP) is a persistent or recurrent pain lasting more than 3 months. It represents a major health issue worldwide, affecting roughly 19% of European and 20% of U.S. adults [1, 2]. CP was originally classified as “nociceptive” (i.e., associated with persistent input from actual or potential tissue damage) and “neuropathic” (due to injury or illness affecting the peripheral or central nervous system) [3, 4]. More recently, a third category of CP conditions—referred to as “nociplastic pain” (NP)—has been operationalized, encompassing syndromes characterized by altered nociceptive processing in the absence of clear evidence of actual or threatened tissue damage, nociceptor activation, or demonstrable neuropathy [3, 5]. The NP is characterized by changes in pain‐related sensory pathways considering both peripheral and central nervous systems, leading to heightened sensitivity toward internal and external stimuli [4, 5].

The NP identifies a spectrum of syndromes with high rates of comorbidity [5] more prevalent in women [6], which includes chronic headache, fibromyalgia, and vulvodynia [4], characterized by distinct overt manifestations. Specifically, chronic headache is defined as “15 or more headache episodes per month for at least three months.” [7–9]. Whereas, patients with fibromyalgia report widespread pain and tenderness as core symptoms of this condition [10]. Nevertheless, individuals with fibromyalgia also experience other distressing somatic and cognitive‐affective symptoms, such as fatigue, sleep disturbances, cognitive impairment, and emotional distress [11–13]. Vulvodynia represents a condition of chronic vulvar pain without an identifiable cause [14]. This condition can have detrimental effects on multiple aspects of the patient’s life, including sexuality and intimacy, subjective psychological well‐being, and general psychosocial adjustment [15, 16]. In line with the concept of an NP spectrum, several studies have reported high co‐occurrence rates among conditions ascribed to it. For instance, the frequency of co‐occurrence of chronic headache among patients with fibromyalgia ranged from 45% to 80% [17, 18], whereas high proportions (20%–36%) of patients with chronic headache met criteria for fibromyalgia [19]. Nevertheless, empirical evidence clarifying clinical characteristics of this complex subgroup of CP conditions remains limited, especially considering factors that may contribute to the co‐occurrence of NP syndromes.

### 1.1. Temperamental and Personality Dimensions in CP Conditions

Temperament refers to automatic associative responses to salient stimuli, which are evident in early infancy and characterized by robust genetic determinants. According to Cloninger [35, 36], temperamental dimensions interplay across the lifespan with other personality traits that capture individual differences in the development of high‐order self‐concept and self‐other relationships (i.e., character). The interplay between low‐order temperamental dimensions and higher‐order personality traits has been further supported by empirical data from other theoretical perspectives [37]. For instance, significant associations between temperamental dimensions (e.g., novelty seeking, harm avoidance) and personality traits such as neuroticism, extraversion, and conscientiousness, which organize mental and behavioral functioning across the lifespan, have been consistently found in several studies [39, 40].

These temperamental and personality traits might represent psychological factors affecting the emergence and clinical course of CP conditions [40]. Empirical data highlighted that subjects with CP conditions were characterized by high levels of harm avoidance (temperament: tendency to be fearful, pessimistic, and sensitive to criticism) in association with low levels of self‐directness (character: difficulty with defining and setting meaningful goals, low motivation, and problems with adaptive coping) [45]. Referring to the five‐factor model of personality, several studies showed consistent results concerning high trait levels of neuroticism and low levels of extraversion among different CP conditions [46, 47].

Despite this evidence, the role of temperament and personality in CP conditions is not fully clarified. Indeed, the vulnerability to the onset of CP conditions is shaped by complex interactions among sensory systems, environmental and psychological factors, and neurobiological mechanisms of pain regulation [41, 85]. Nevertheless, hypersensitivity to environmental stimuli (e.g., light or sound) has been viewed as a temperamental dimension linked to “sensory processing sensitivity” [5, 41], which is widely considered a core feature of conditions ascribed to the NP spectrum. Specifically, the link between sensory processing sensitivity and clinical conditions characterized by NP has been supported by empirical evidence that demonstrated how a heightened processing of environmental stimuli leads to a distressing perception of the external world and chronic stress [44], which are considered relevant precipitant factors for the emergence of CP syndromes [82]. The sensory processing sensitivity refers to genetically based individual differences in sensitivity to internal and external stimuli [42, 43]. Individuals with high levels of sensory processing sensitivity report a heightened sensory sensitivity and a more detailed processing of salient stimuli. Accordingly, it has been suggested that a lower sensory threshold might represent an intermediate phenotype capturing temperamental/genetic and neurobiological risk factors for the emergence of CP [41].

Notably, it has been suggested that sensory processing sensitivity temperamental dimensions are related to, albeit distinct from, other high‐order personality traits [42]. Specifically, a meta‐analytic review [43] highlighted small‐to‐moderate associations between SPS with introversion, neuroticism, openness, behavioral inhibition, and negative affectivity, suggesting that SPS temperamental traits could not fully overlap with these personality traits. Nevertheless, no studies have clarified how SPS temperamental and personality traits may be involved in different CP conditions. Moreover, no studies have explored whether specific patterns of associations among these dimensions might discriminate distinct CP conditions.

### 1.2. The Current Study

Departing from the previously mentioned evidence and considerations, the current study aims at examining implications of sensory processing sensitivity, temperamental and other maladaptive personality traits among different CP groups ascribed to the NP spectrum, including chronic headache, fibromyalgia, vulvodynia, and patients with comorbid CP conditions. Particularly, levels of sensory processing sensitivity features and maladaptive personality traits were compared between a group composed of all conditions ascribed to the NP spectrum and healthy controls (HCs). Furthermore, we explored the structure of associations among sensory processing sensitivity, temperamental and maladaptive personality traits in order to clarify how they might interplay with each other in CP conditions compared to HCs. To do so, a network analysis approach was applied. The main advantage of using network analysis to study the interrelationships among psychological variables is that it allows for the robust identification of the most influential dimensions within a set of variables characterizing a specific clinical group [83]. Therefore, network analysis represents an innovative, data‐driven statistical approach for capturing the core features of clinical conditions [84].

A second step of analysis was also conducted, focusing on temperamental and maladaptive personality trait profiles that might quantitatively discriminate CP conditions from each other and from HCs. Moreover, network analysis techniques were applied to explore qualitative differences in the organization of temperamental and maladaptive personality traits across each CP condition. This was aimed at determining whether each CP condition—characterized by different symptomatic manifestations—could be organized around distinct organizations of temperamental and maladaptive personality traits.

## 2. Materials and Methods

### 2.1. Procedures

This study adopted a cross‐sectional research design. Therefore, the data collected reflect a static profile of temperament and maladaptive personality traits, without providing information about their development across the lifespan. An Italian cohort of 1237 participants was recruited through a snowball sampling technique supported by Italian patients’ associations of individuals affected by chronic headache, fibromyalgia, and vulvodynia, which disseminated the web survey across their institutional websites and various social media platforms such as Facebook, Instagram, X, and LinkedIn. The survey was administered using Google Forms. Data collection ranged from April 2023 to January 2024. Participants provided informed consent prior to participating in the survey. They self‐reported information regarding their CP diagnoses, the year of diagnosis, and the healthcare professional or medical facility responsible for the diagnosis. Anonymity was ensured throughout the survey process, and participants did not receive any form of compensation for their participation. Ethical clearance for the project was obtained from the ethical committee of the D. D C. P. and H. S. at S. U. Eligible participants were required to self‐identify as cisgender women, be aged 18 years or older, be proficient in Italian, and have received a diagnosis of chronic headache, fibromyalgia, and/or vulvodynia from a specialist physician (neurologists, rheumatologists, and gynecologists) at least six months prior. Ninety‐three responses (7.52%) were excluded from the analysis due to noncompliance with inclusion criteria or duplicate entries, resulting in a total group of 1144 women. Sociodemographic characteristics of the participants included in this study are summarized in Table 1.

**Table 1:** Sociodemographic data and description of the participants (*n* = 1144).

	Total group (*n* = 1144)	Chronic headache (CH) (*n* = 222)	Fibromyalgia (FM) (*n* = 201)	Vulvodynia (VU) (*n* = 221)	Complex (Com) (*n* = 359)	Healthy controls (HC) (*n* = 141)	Significance
	M (SD)	M (SD)	M (SD)	M (SD)	M (SD)	M (SD)	*F*
Age	40.71 (13.31)	39.6 (12.20)	46.9 (12.20)	31.9 (10.60)	44.7 (12.50)	36.70 (13.70)	55.9 *df = *4.11 *p* < 0.001

	***n* (%)**	***n* (%)**	***n* (%)**	***n* (%)**	***n* (%)**	***n* (%)**	**Chi-squared**

Sexual orientation							27.10 df = 12 *p* = 0.007
Heterosexual	1052 (92.00)	200 (90.10)	194 (96.50)	197 (89.10)	336 (93.60)	125 (88.70)	
Bisexual, pansexual, or polisexual	67 (5.90)	14 (6.30)	3 (1.50)	15 (6.80)	21 (5.80)	14 (9.90)	
Lesbian	15 (1.30)	4 (1.80)	4 (2.00)	5 (2.30)	0	2 (1.40)	
Asexual spectrum	10 (0.90)	4 (1.80)	0	4 (1.80)	2 (0.60)	0	
Civil status							135.00 df = 16 *p* < 0.001
Single	439 (38.40)	92 (41.40)	46 (22.90)	123 (55.70)	102 (28.40)	76 (53.90)	
Married/civil union	427 (37.30)	84 (37.80)	106 (52.70)	36 (16.30)	167 (46.50)	34 (24.10)	
Separated/divorced	90 (7.90)	14 (6.30)	18 (9.00)	7 (3.20)	42 (11.70)	9 (6.40)	
Widowed	9 (0.80)	0	2 (1.00)	1 (0.50)	5 (1.40)	1 (0.70)	
Cohabitant	179 (15.01)	32 (14.40)	29 (14.40)	54 (24.40)	43 (12.00)	21 (14.90)	
Current relational status							
Single	243 (21.20)	39 (17.60)	42 (20.90)	41 (18.60)	83 (23.10)	38 (27.00)	
Monogamous couple	886 (77.40)	176 (79.30)	156 (77.60)	179 (81.00)	273 (76.00)	102 (72.30)	
Polyamorous/nonmonogamous relationship	15 (1.30)	7 (3.20)	3 (1.50)	1 (0.50)	3 (0.80)	1 (0.70)	
Polyamorous/nonmonogamous relationship	15 (1.30)	7 (3.20)	3 (1.50)	1 (0.50)	3 (0.80)	1 (0.70)	
Education degree							
Primary school	1 (0.10)	0	0	1 (0.50)	0	0	
Middle school	77 (6.79)	9 (4.10)	23 (4.10)	4 (1.80)	35 (9.7)	6 (4.30)	
High school	472 (41.30)	88 (39.60)	99 (49.30)	81 (36.70)	169 (47.10)	35 (24.80)	
Degree	445 (38.90)	94 (42.30)	65 (32.30)	105 (47.50)	114 (31.80)	67 (47.50)	
Postdegree	149 (13.00)	31 (14.00)	14 (7.00)	30 (13.60)	41 (11.40)	33 (23.40)	
Work status							
Unemployed	211 (18.40)	49 (22.10)	43 (21.40)	23 (10.40)	83 (23.10)	13 (9.20)	102 df = 12 *p* = < 0.001
Student	159 (13.90)	31 (14.00)	7 (3.50)	61 (27.60)	27 (7.50)	33 (23.40)	
Employed	717 (62.70)	136 (61.30)	133 (66.20)	134 (60.60)	227 (63.20)	87 (61.70)	
Retired	57 (5.00)	6 (2.70)	18 (9.00)	3 (1.40)	22 (6.10)	8 (5.70)	

### 2.2. Participants

The mean age of participants was 40.71 years [14, 18–74]. A large portion of participants attained moderate levels of education, corresponding to completion of a high school diploma or university degree. All participants had received a diagnosis of CP between 1982 and 2022 from neurologists, rheumatologists, and gynecologists. Participants were categorized into five groups: chronic headache, fibromyalgia, vulvodynia, patients with comorbid CP conditions—when participants had two or more CP diagnoses—and HCs—women who reported no history of CP related to the three diagnoses considered for the study.

### 2.3. Measures

The study administered a battery of self‐report instruments to investigate sensory processing sensitivity and maladaptive personality traits aligned with the objectives of the research project. The completion time for the assessment was approximately 30 min. The questionnaires administered were the following:

*Sociodemographic Questionnaire*—Participants were requested to complete a concise sociodemographic survey aimed at gathering general information, including age, gender, sexual orientation, marital status, relationship status, level of education, employment status, socioeconomic status, ethnicity, residential location, and pertinent details regarding the diagnosis of CP conditions.

*Highly Sensitive Person Scale* [42, 91]. An Italian translation of the 12‐item Highly Sensitive Person Scale was administered [48]. This questionnaire was designed to assess the trait of sensory processing sensitivity. Factor analyses suggested three subscales: (a) Aesthetic Sensitivity (AES), which captures aesthetic awareness (e.g., being deeply moved by the arts and music); (b) Low Sensory Threshold (LST), which reflects unpleasant sensory arousal to external stimuli (e.g., a negative reaction to bright lights and loud noises); and (c) Ease of Arousal (EOE), which describes the tendency to be easily overwhelmed by external and internal stimuli (e.g., experiencing a negative response to “having many things going on at once” or performing a task worse when being observed). Respondents rated these items on a 7‐point Likert scale, ranging from 1 (*strongly disagree*) to 7 (*strongly agree*). The scores were calculated to obtain a measure of the sensitivity of the different domains, with higher scores indicating higher levels of sensitivity. The original validation studies [42, 91] reported robust values of internal consistency (i.e., 0.85 ≤ *α* ≤ 0.87). The Italian translation [48] showed good internal consistency (*α* = 0.75). An adequate internal consistency was replicated in the current study for each subscale within groups of interest (AES: 0.79 ≤ *α* ≤ 0.81; EOE: 0.82 ≤ *α* ≤ 0.86; LST: 0.84 ≤ *α* ≤ 0.88).

*Personality Inventory for DSM-5–Short Form* [49]. The Italian version of the Personality Inventory for DSM‐5–Short Form was administered [49]. It consists of 25 items. Each item is rated on a 4‐point Likert scale (0: *very false* or *often false*–3: *very true* or *often true*). This instrument comprehensively evaluates the 5 high‐order maladaptive domains of personality according to the DSM‐5 section III: (i) Negative Affectivity (NA): frequent and intense experiences of a wide range of negative emotions (e.g., anxiety, depression, guilt/shame, worry, anger) and related behavioral (e.g., self‐harm)/interpersonal (e.g., dependency) manifestations; (ii) Detachment (DE): avoidance of socioemotional experiences (e.g., withdrawal from interpersonal interactions), as well as restricted affective experiences and expressions (e.g., limited hedonic capacity); (iii) Antagonism (AN): behaviors that suggest an exaggerated sense of self‐importance and a concomitant expectation of special treatment, together with a callous antipathy toward others (e.g., unawareness of others’ needs and feelings), and a readiness to use others in the service of self‐enhancement; (iv) Disinhibition (DI): an orientation toward immediate gratification, leading to impulsive behaviors driven by current thoughts, feelings, and external stimuli, without regard for past learning or consideration of future consequences; and (v) Psychoticism (PSY): manifestations of a wide range of culturally incongruent odd, eccentric, or unusual behaviors and cognitions, including both process (e.g., unusual perception, dissociation) and content (e.g., beliefs). The total score of each domain is derived by computing the sum of the scores of items. Consequently, higher scores indicate a greater expression of these maladaptive personality traits. The validity and reliability (i.e., 0.85 ≤ *α* ≤ 0.89) of PID‐5‐SF domains were demonstrated in the original validation studies [89, 90]. Acceptable internal consistency indices were replicated in the current study across all groups (NA: 0.73 ≤ *α* ≤ 0.75; DE: 0.71 ≤ *α* ≤ 0.74; AN: 0.70 ≤ *α* ≤ 0.72; DI: 0.72 ≤ *α* ≤ 0.79; PSY: 0.71 ≤ *α* ≤ 0.75).

### 2.4. Statistical Analysis

Power analysis was conducted using GPower 3.1 [50]. According to the aims of the study, the power analysis for estimating MANOVA effects was set using the following parameters: (i) number of groups: 5; (ii) maximum number of variables: 6 (i.e., 5 maladaptive personality traits, 1 covariate); (iii) *α* corrected applying Bonferroni procedures for post hoc comparisons (*N* = 9): *α* = 0.005; (iv) 1‐*β* = 0.995; and (v) a small effect size (*f* ^2^ *= *0.02). Results showed a minimum sample equal to 744 subjects.

SPSS 22, JASP 0.16.3, and RStudio {bootnet}, {qgraph}, {psych}, {reshape2} packages were used to analyze data. The ANOVA and *χ* ^2^ test were conducted in order to evaluate differences among CP groups and HCs in the distribution of socio‐demographic variables (e.g., age, sexual orientation). In case of significant differences, variables were introduced as covariates in statistical models for testing the hypotheses of the study. Specifically, MANCOVA analyses were applied to explore significant differences between CP groups and HCs with respect to sensory processing sensitivity, temperamental and maladaptive personality traits. Referring to the hypotheses of the study, post hoc comparisons using Bonferroni correction were estimated to identify specific profiles differentiating specific CP conditions from each other and from HCs. Partial eta‐squared ($\eta_{p}^{2}$) was used as an effect size measure for MANCOVA analyses. According to Cohen’s guidelines [92], values of 0.01, 0.06, and 0.14 indicate small, medium, and large effect sizes, respectively. Cohen’s *d* [92] was estimated as an effect measure for post hoc comparisons among specific CP conditions and HCs. Values of 0.20, 0.50, and 0.80 indicate small, medium, and large effect sizes, respectively [92].

Network analysis was conducted to examine the structure of relationships among SPS temperamental and maladaptive personality traits within each group of interest. Network analysis allows us to estimate 3 main indexes: (i) *closeness* (the inverse of the sum of all shortest paths from the node of interest to all other nodes). A node with high closeness suggests how quickly that parameter can be affected by changes in any part of the network and how quickly it can affect changes in other parts of the network; (ii) *betweenness* (the number of shortest paths that pass through the node of interest) indicates how important a specific node is for connecting other two nodes of the network; and (iii) *strength* (the sum of the absolute input weights of that node) represents how strongly a node is directly connected to other nodes, suggesting the degree of importance of a node for the entire network.

The EBICglasso algorithm (i.e., computing a sparse Gaussian graphical model with the graphical lasso) [51] was used for calculating the indexes mentioned above. The tuning parameter was set at 0.5 using the Extended Bayesian Information Criterion (EBIC) [52, 53]; this 0.5 indicates that more parsimonious models with fewer edges are preferred. This method provides a network of partial correlation coefficients with a limited number of spurious edges [52, 53]. Centrality indices were plotted using standardized z‐scores to facilitate interpretation. *Network accuracy* [52] was computed employing a nonparametric bootstrap approach. We estimated edge weights accuracy at 95% confidence intervals (CIs) by sampling the data 2000 times (with replacement), thereby generating a distribution of edge weights. The difference tests among centrality values were also estimated using the nonparametric bootstrapped difference test. Ultimately, the *correlation stability coefficient* (CS‐C) was estimated for each centrality index in order to test the reliability of network analysis results. Values equal to or greater than 0.25 were considered acceptable [52].

## 3. Results

Significant differences were found in age (*F*_(4, 1139)_ = 55.90; *p* < 0.001). Bonferroni’s post hoc comparisons revealed that patients ascribed to the fibromyalgia group and those with comorbid CP conditions were significantly older than other groups. Furthermore, significant differences were observed among groups with respect to sexual orientation, civil status, education, and employment status. It should be noted that age may significantly contribute to the previous differences in distribution of socio‐demographic variables. Indeed, older participants could be more frequently married, might have higher educational attainment, and may be more often employed or retired. These considerations supported the inclusion of age as the only covariate in the MANOVA analysis. Table 2 summarizes descriptive and MANCOVA results considering SPS and maladaptive personality traits.

**Table 2:** Comparisons among groups of interest with respect to temperamental and maladaptive personality traits.

	CH (*n* = 222) M (SE)	FM (*n* = 201) M (SE)	VU (*n* = 221) M (SE)	COMPLEX (*n* = 359) M (SE)	CPG (*n* = 1003) M (SE)	HC (*n* = 141) M (SE)	Multivariate effects of CPG vs. HCs	Comparison of CPG vs. HC	Multivariate effects of CP‐related conditions vs. HCs	Post hoc comparison of CP conditions vs. HCs
*Temperamental dimensions*										
Aesthetic Sensitivity	5.45 (0.07)	5.37 (0.08)	5.56 (0.08)	5.44 (0.06)	5.45 (0.03)	5.56 (0.09)	*F*_(3, 1139)_ = 20.45^∗∗∗^ $\eta_{p}^{2}$ *= *0.05	—	*F*_(12, 3414)_ = 14.13^∗∗∗^ $\eta_{p}^{2}$ = 0.05	—
Low Sensitivity Threshold	5.25 (0.09)	4.9 (0.09)	4.30 (0.09)	5.31 (0.07)	5.01 (0.04)	4.19 (0.11)		HC < CPG^∗∗∗^		HC < CH^∗∗∗^, FM^∗∗∗^, COMPLEX^∗∗∗^ VU < CH^∗∗∗^, FM^∗∗∗^, COMPLEX^∗∗∗^ FM < COMPLEX^∗^
Ease of Excitation	5.37 (0.09)	5.48 (0.10)	5.19 (0.10)	5.56 (0.07)	5.42 (0.03)	4.83 (0.11)		HC < CPG^∗∗∗^		HC < CH^∗∗^, FM^∗∗∗^, COMPLEX^∗∗∗^ VU < COMPLEX ^∗∗^

*Maladaptive personality traits*										
Psychoticism	4.28 (0.21)	4.40 (0.22)	4.41 (0.22)	4.93 (0.16)	4.56 (0.10)	3.51 (0.26)	*F*_(5, 1137)_ = 7.52^∗∗∗^ $\eta_{p}^{2}$ = 0.03	HC < CPG^∗∗∗^	*F*_(20, 4548)_ = 3.20^∗∗∗^ $\eta_{p}^{2}$ *= *0.01	HC < FM^∗^, COMPLEX^∗∗∗^ CH < COMPLEX^∗^
Disinhibition	3.36 (0.19)	3.48 (0.21)	3.29 (0.20)	3.60 (0.15)	3.47 (0.09)	3.07 (0.24)		—		—
Antagonism	3.04 (0.16)	2.86 (0.17)	3.06 (0.16)	3.13 (0.12)	3.04 (0.07)	2.79 (0.20)		—		—
Detachment	4.55 (0.19)	4.93 (0.21)	4.41 (0.20)	5.12 (0.16)	4.81 (0.09)	3.71 (0.26)		HC < CPG^∗∗∗^		HC < FM^∗∗∗^, COMPLEX^∗∗∗^ CH, VU < COMPLEX^∗^
Negative Affectivity	7.55 (0.21)	7.35 (0.23)	7.41 (0.22)	7.71 (0.17)	7.54 (0.10)	6.01 (0.27)		HC < CPG^∗∗∗^		HC < CH^∗∗∗^, FM^∗∗∗^, VU^∗∗∗^, COMPLEX^∗∗∗^

*Note:* This reported marginal means and related standard error estimated including age as a covariate; FM = fibromyalgia; M = mean; VU = vulvodynia.

Abbreviations: CH = chronic headache, CPG = chronic pain group, HC = healthy control, SE = standard error.

^∗^ *p* < 0.05.

^∗∗^ *p* < 0.01.

^∗∗∗^ *p* < 0.001.

### 3.1. Temperamental and Maladaptive Personality Traits Associated With CP

The analysis found a significant multivariate effect of the CP group for levels of sensory processing sensitivity and temperamental traits. Specifically, the CP group showed higher levels of LST (*F*_(4, 1139)_ = 44.94; *p* < 0.001; $\eta_{p}^{2}$ = 0.04) and EOE (*F*_(4, 1139)_ = 26.56; *p* < 0.001; $\eta_{p}^{2}$ = 0.02) than HCs. The effect sizes were small to moderate (LST: *d = *0.68 [0.50–0.86]; EOE: *d = *0.45 [0.27–0.63]). Similarly, a multivariate effect was found considering maladaptive personality traits. Particularly, the CP group highlighted significantly higher levels of NA (*F*_(4, 1139)_ = 32.63; *p* < 0.001; $\eta_{p}^{2}$ = 0.03; *d = *0.49 [0.31–0.86]), DE (*F*_(4, 1139)_ = 19.31; *p* < 0.001; $\eta_{p}^{2}$ = 0.02; *d = *0.39 [0.22–0.57]), and PSY (*F*_(4, 1139)_ = 16.56; *p* < 0.001; $\eta_{p}^{2}$ = 0.01; *d = *0.32 [0.14–0.49]) than HCs. These differences were small to moderate.

**Figure 1:**
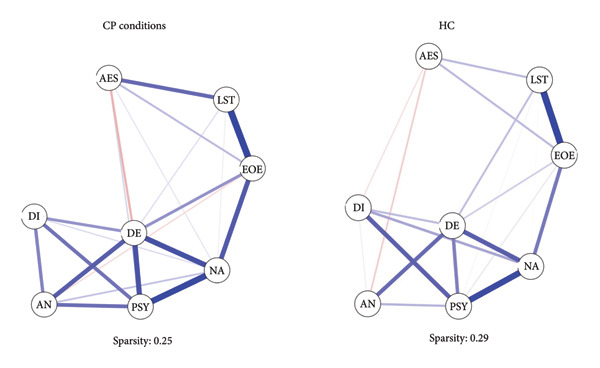
Network analysis of temperamental and personality traits. AES = Aesthetic Sensitivity; AN = Antagonism; DE = Detachment; DI = Disinhibition; EOE = Ease of Excitation; LST = Low Sensitivity Threshold; NA = Negative Affectivity; PSY = Psychoticism.

Looking at network analysis (see Figure 1), the DE maladaptive personality domain represented the node with the highest centrality indexes (i.e., closeness, betweenness, and strength) within the CP group. Closeness (CS‐C: 0.75) and strength (CS‐C: 0.75) indexes showed a high robustness. On the contrary, betweenness did not reach an acceptable value of CS‐C (0.05); therefore, this value was not reliable. Testing the difference among centrality values (see supporting information: Figure 1s), DE, NA, and PSY showed no significant differences in degree of centrality values. Whereas, these maladaptive personality domains highlighted significantly higher degree of centrality values than the other temperamental and maladaptive personality dimensions. Closeness values of DE, NA, and PSY were not statistically different from each other, but these indexes were higher than the remaining temperamental and maladaptive personality trait dimensions.

**Figure 2:**
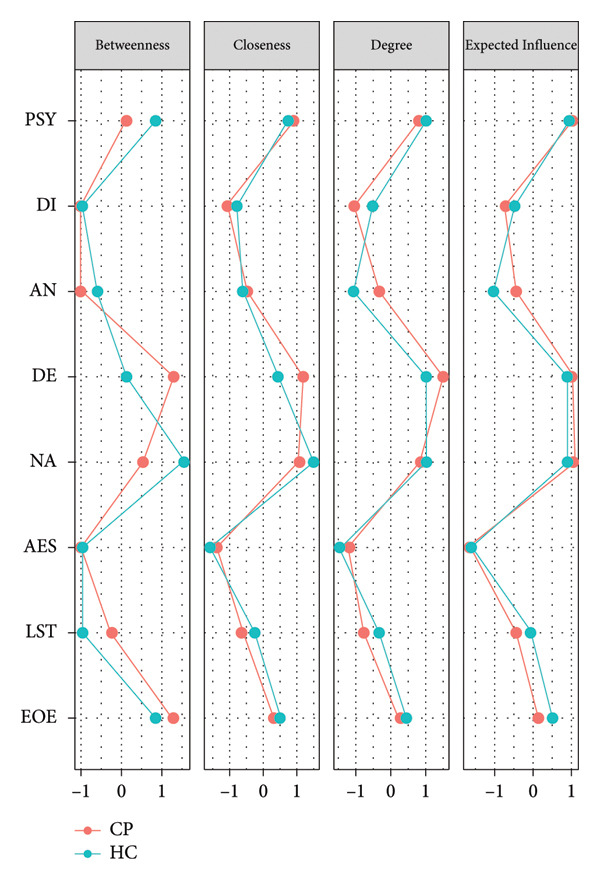
Centrality plots for CP conditions and HC. AES = Aesthetic Sensitivity; AN = Antagonism; DE = Detachment; DI = Disinhibition; EOE = Ease of Excitation; LST = Low Sensitivity Threshold; NA = Negative Affectivity; PSY = Psychoticism.

Referring to HCs, NA, DE, and PSY maladaptive domains showed the highest values of degree of centrality, but they were not different from the EOE temperamental dimension. Looking at CS‐Cs, closeness (0.28) and strength (0.44) showed acceptable values. Whereas, the CS‐C of betweenness (0.05) suggested that this index was not reliable. Therefore, this centrality index was not considered. Contrary to CP conditions, the NA represented the node with the highest values of closeness. The NA closeness value was significantly higher than AN/DI maladaptive personality traits and AES/LST temperamental traits. On the contrary, closeness values of NA, DE, PSY, and EOE did not significantly differ from each other (see supporting information for results of difference tests: see Figure 2s). Figure 2 shows centrality plots and network accuracy analysis. Table 1s reports associations among temperamental and maladaptive personality traits in CP and HC groups.

### 3.2. Temperamental and Maladaptive Personality Traits Dimensions Within Specific CP Conditions

Table 2 summarizes MANCOVA results among groups of interest. Considering SPS temperamental traits, the analysis found a multivariate effect of groups of interest. Specifically, LST was a shared temperamental trait among chronic headache and fibromyalgia groups together with patients affected by comorbid CP conditions, and it significantly differentiated them from HCs—chronic headache: *d = *0.91 [0.68–1.13], fibromyalgia: *d = *0.76 [0.54–0.98], comorbid CP conditions: *d = *1.02 [0.82–1.23]—and patients with vulvodynia—chronic headache: *d = *0.80 [0.61–0.99], fibromyalgia: *d = *0.68 [0.49–0.88], comorbid CP conditions: *d = *0.94 [0.76–1.11]. Interestingly, LTS also distinguished patients with comorbid CP conditions from those with FM, even though the effect size was small (*d = *0.24 [0.06–0.41]). Similar findings were detected considering the EOE subscale. Indeed, this differentiated CP conditions from HCs—chronic headache: *d = *0.40 [0.19–0.61], fibromyalgia: *d = *0.45 [0.24–0.67], and comorbid CP conditions: *d = *0.55 [0.35–0.75]. Patients with comorbid CP conditions also highlighted significantly higher levels than patients with vulvodynia (*d = *0.24 [0.07–0.41]).

Looking at maladaptive personality traits, results showed a multivariate effect of groups on levels of PID‐5 domains. Particularly, the NA was a shared maladaptive trait among CP conditions that significantly differentiated them from HCs—chronic headache: *d = *0.48 [0.26–0.69], vulvodynia: *d = *0.49 [0.28–0.71], fibromyalgia: *d = *0.40 [0.18–0.62], patients with comorbid CP conditions: *d = *0.54 [0.34–0.74]. The DE and PSY factors highlighted the highest levels in patients with comorbid CP syndromes, and they differentiated this group from chronic headache and vulvodynia but not from fibromyalgia. The effect sizes of these contrasts were small.

### 3.3. Network Analysis for Each CP Condition

**Figure 3:**
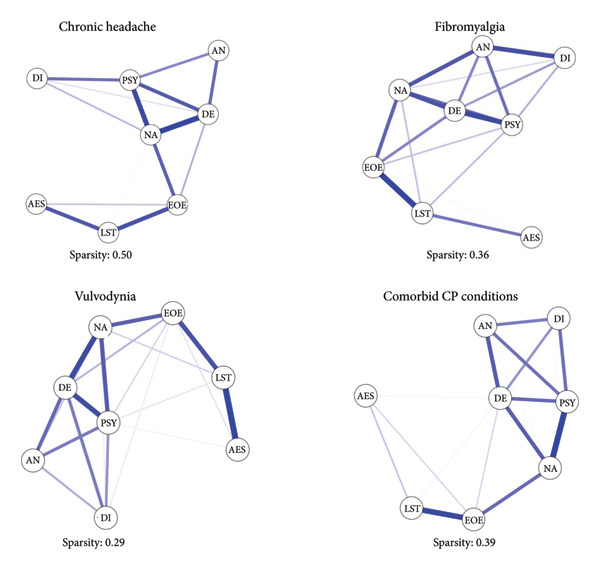
The structure of the network for each CP condition. AES = Aesthetic Sensitivity; AN = Antagonism; DE = Detachment; DI = Disinhibition; EOE = Ease of Excitation; LST = Low Sensitivity Threshold; NA = Negative Affectivity; PSY = Psychoticism.

**Figure 4:**
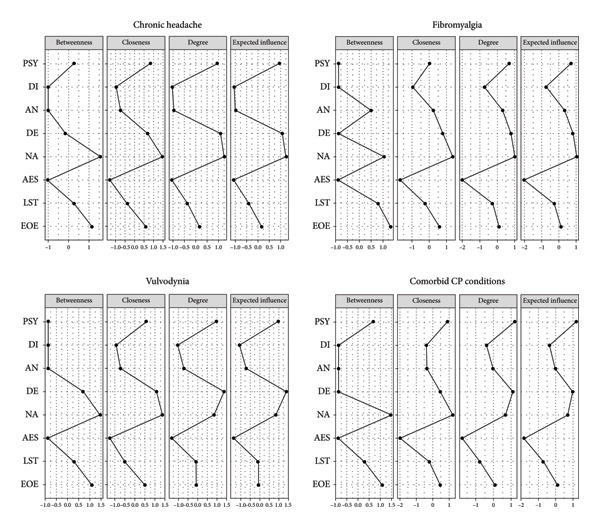
Centrality indexes for each CP condition. AES = Aesthetic Sensitivity; AN = Antagonism; DE = Detachment; DI = Disinhibition; EOE = Ease of Excitation; LST = Low Sensitivity Threshold; NA = Negative Affectivity; PSY = Psychoticism.

Figure 3 depicts the structure of the network of temperamental and maladaptive personality traits for each CP condition. Figure 4 summarizes centrality indexes for each specific CP condition. Tables 2s–5s report a detailed description of associations among the investigated dimensions within each clinical group.

Looking at patients with chronic headache, the CS‐Cs supported the reliability of centrality measures—betweenness (0.36), closeness (0.52), and strength (0.59). Similarly, the vulvodynia group showed adequate CS‐Cs of centrality indexes—betweenness (0.33), closeness (0.51), and strength (0.59). Conversely, the analyses confirmed the robustness of CS‐Cs only for closeness and strength in the fibromyalgia group (0.43; 0.59) and among patients with comorbid CP conditions (0.44; 0.75). Accordingly, values of betweenness in these groups were not considered.

Considering strength values, the most influential nodes of the network associated with all CP conditions were NA, DE, and PSY. Looking at SPS temperamental traits, the EOE dimension seemed to be the most representative for all CP conditions. Whereas, DE and PSY betweenness values were different among CP‐related conditions, supporting specific network structures for each condition (see Figure 3). Particularly, the chronic headache was characterized by a high value of PSY betweenness; on the other hand, vulvodynia highlighted a null value of betweenness for this maladaptive personality trait. Whereas, patients with vulvodynia highlighted higher DE betweenness values if compared to the other clinical groups, which were characterized by null betweenness values for this maladaptive personality trait. Supporting information (see Figures 3s–6s) report results of network accuracy and difference test analyses.

## 4. Discussion

The current study sought to clarify implications of sensory processing sensitivity, temperamental and maladaptive personality traits for different CP conditions ascribed to the NP spectrum. Accordingly, the current study explored which SPS temperamental and maladaptive personality traits might characterize CP conditions, together with how these psychological factors might specifically interplay with each other within each CP syndrome. This was evaluated in order to highlight whether distinct CP conditions could be linked to specific patterns of organization of personality traits and related psychological processes. The analyses showed 4 main findings:

i. CP diseases were characterized by higher levels of EOE and LST together with NA, DE, and PSY compared to HCs. The extent of these differences was small to moderate;
ii. NA, DE, and PSY were the central nodes of the network of temperamental and maladaptive personality traits, especially considering closeness and strength indexes. The DE domain differentiated CP groups from HCs. Particularly, DE was the central node of the network (i.e., strength). Whereas, the HC group showed that the NA represented the most central node;
iii. Distinct subgroups of CP conditions reported the same higher levels of EOE, LST, and NA than HCs. Whereas, higher levels of DE and PSY maladaptive personality traits differentiated patients with comorbid CP conditions from individuals with a single CP syndrome;
iv. All CP conditions showed that NA, DE, and PSY maladaptive traits were the most central nodes of the network. Nevertheless, chronic headache and vulvodynia differed from each other with respect to the role of PSY as a bridge (i.e., betweenness values) for the other nodes of the network. On the contrary, the betweenness values of patients with fibromyalgia and affected by comorbid CP conditions were not robust enough to be interpreted.

Departing from sensory processing sensitivity, temperamental traits, and related effect sizes of MANCOVA contrast analyses, the current results are fully in line with theoretical frameworks and empirical evidence [4, 5, 41] that have supported key implications of sensory processing sensitivity for clinical features of different CP conditions. Referring to current findings, the most relevant dimensions related to sensory processing sensitivity involved in CP conditions were the tendency to experience unpleasant sensory arousal in response to external stimuli (i.e., LST; quality of reactions) together with a hyper‐reactivity toward internal and external salient situations (i.e., EOE; intensity of responses). These temperamental traits seem to partially overlap with central sensitization [54, 55], which is considered a core pathophysiological mechanism involved in the development of NP [56, 57]. The small‐to‐moderate differences observed in the current large sample suggest the clinical relevance of these temperamental traits for the assessment of psychological functioning of CP conditions and the identification of key features that should be addressed by different psychotherapeutic approaches. Accordingly, clinical psychological interventions specifically developed to target emotional reactivity (e.g., mindfulness‐based, experiential/psychodynamic, and emotion‐regulation interventions) [93, 94] should be considered the first choice to treat CP conditions.

Regarding maladaptive personality traits, the CP group highlighted significantly higher levels of NA, which may represent a dimension involved in the high co‐occurrence rates revealed between these syndromes and internalizing psychopathological disorders (e.g., depression, anxiety, and PTSD), which are characterized by core emotional dysfunctions [21, 22]. This finding also corroborates the central role of altered emotional functioning for clinical features characterizing CP conditions and related considerations concerning the choice of specific therapeutic interventions developed to improve affective regulation [95].

Furthermore, the DE dimension significantly, albeit modestly, differentiated the CP group from HCs, and it represented the most central node of the network of temperamental and maladaptive personality traits of this clinical group. Interestingly, a meta‐analysis [22] showed that the DE maladaptive domain could be viewed as a key feature specifically associated with different personality disorders (e.g., avoidant personality disorder) and personality problems linked to interpersonal functioning (e.g., connection with others, desire, and capacity for closeness) [58]. Accordingly, the high levels of DE reported by patients with CP conditions may be consistent with empirical findings demonstrating their comorbidity rates with personality disorders [59, 60]. Furthermore, this result could be associated with well‐documented interpersonal difficulties (e.g., social isolation; unsatisfactory relationships) reported by patients with different CP conditions [61]. Moreover, it has been suggested that interpersonal dysfunctions significantly contribute to the severity of pain symptoms [62, 63], and they are associated with psychopathological phenomena (e.g., depression and anxiety) among patients with various CP conditions [64]. According to this evidence, effective and comprehensive psychotherapeutic interventions for CP conditions should consider interpersonal functioning as one of the primary targets of treatment.

Enriching the profile of personality traits of the CP group, PSY played a significant, albeit marginal, role in these syndromes. Referring to the DSM‐5 conceptualization [49], the PSY domain captures the tendency to manifest eccentric behaviors and unusual beliefs together with perceptual dysregulation and dissociative experiences [65, 66]. On the one hand, there is no robust evidence regarding clinical implications of psychotic‐like phenomena among patients with CP. On the other hand, dissociation—*disruption of and/or discontinuity in the normal integration of consciousness, memory, identity, emotion, perception, body representation, motor control, and behavior* [99]—and related processes (e.g., fragmentation of experiences, inability to access information or to control mental functions, detachment from emotional experiences) [65, 66] has been recurrently documented among patients with CP [23–26, 32, 96–98]. Following the nosography of dissociation [67–69] and empirical studies previously mentioned, positive (e.g., pain) and negative (e.g., anesthesia) somatoform dissociative symptoms [68, 69] might be considered the most relevant for CP conditions, especially considering their maladaptive function of regulation of affective states [70, 71], which shows a basic alteration as demonstrated by current studies. Furthermore, higher levels of PSY and related dissociative phenomena might be associated with adverse and traumatic experiences [72, 73] that have been recurrently documented in these clinical populations [74]. Nevertheless, the link between the DSM‐5 PSY maladaptive trait, dissociation, and traumatic experiences should be demonstrated through future studies among clinical populations of patients with CP. However, psychological interventions for CP might consider dissociation as a secondary target.

Results of network analysis showed that the previously mentioned maladaptive personality traits, rather than sensory processing sensitivity temperamental dimensions, should be considered as the most relevant for describing the core psychological features of individuals affected by CP conditions. Accordingly, it could be possible to conclude that the maladaptive psychological functioning characterizing CP conditions might overlap with psychopathological disorders ascribed to the internalizing spectrum, namely a clinical group characterized by inwardly directed distress, including pervasive negative affect, heightened self‐focused processing, and difficulties with emotion regulation [22]. Specifically, DE was the most central for the personality traits network of CP conditions. Referring to empirical findings concerning the implications of DE [22, 58–60], interpersonal functioning organized around the avoidance of socioemotional experiences (e.g., withdrawal from interpersonal interactions) together with the lack of hedonic feelings linked to them might be considered one of the most representative personality difficulties of individuals with CP. This might reflect the well‐documented low levels of quality of life reported by individuals with these syndromes, especially considering the relational satisfaction [75]. The central role of DE could be also in line with clinical findings that showed significant improvements of pain symptoms and other symptomatic outcomes (e.g., depression) when interventions target interpersonal functioning of patients with CP [76].

Considering distinct subgroups of CP conditions, results might suggest that these syndromes are characterized by common temperamental dimensions capturing alterations of emotional responsiveness toward internal and external stimuli (i.e., EOE and LST) together with an overall tendency to react to them with negative affective states (i.e., NA). This evidence is fully in line with the hypothesis concerning a cluster of CP conditions characterized by similar core features related to alterations of emotional processing systems, which are manifested through central sensitization symptoms [11]. Nonetheless, the group composed of patients affected by comorbid CP conditions, especially fibromyalgia and chronic headache, showed higher levels of DE and PSY than the other clinical groups. Taking into account considerations concerning associations between DE and PSY with maladaptive interpersonal functioning and dissociative phenomena, it could be possible to conclude that the group of patients affected by comorbid CP conditions (COMPLEX group) could be characterized by more severe disorganization of the self [77, 78]. This consideration based on empirical evidence from neuroscience and clinical data could support the hypothesis that individuals who suffer from multiple CP conditions might represent a distinct subgroup of CP syndromes characterized by a developmental history constellated from repeated adverse and traumatic experiences [79, 80], especially relational ones. Nonetheless, this hypothesis should be empirically tested with future studies that systematically assess traumatic experiences and related dissociative reactions together with their implications for interpersonal functioning using a longitudinal perspective.

Despite this evidence, some limitations must be discussed. First, the cross‐sectional nature of the research design did not allow for determining causal relationships of sensory processing sensitivity, temperamental and maladaptive personality traits on the development and maintenance of different CP conditions. Therefore, future longitudinal studies should be carried out in order to empirically evaluate developmental pathways of CP conditions. Precisely, longitudinal research should test mediational and path analysis models to evaluate differential effects of NA, DE, and PSY on key clinical features of specific CP conditions departing from shared sensory processing sensitivity, temperamental vulnerabilities.

On the one hand, it is well‐established that the investigated CP conditions are more prevalent in women [6]. On the other hand, it could be useful to replicate the current findings among samples composed of men, especially considering chronic headache, fibromyalgia, and patients with comorbid CP conditions, in order to generalize the current results. Moreover, future studies should corroborate the current evidence within clinical settings, including treatment‐seeking patients with different CP conditions. This should address a limitation linked to the self‐report diagnoses of CP conditions. Furthermore, self‐reported diagnoses of specific CP conditions based on criteria that have changed in the last 40 years (e.g., fibromyalgia, chronic headache) represented an additional limitation of the study that might affect levels of temperamental and maladaptive personality traits reported by subjects included in the current research. An additional limitation of the study might be the lack of evaluation of the severity of psychopathological symptoms and levels of mental/physical pain at the moment of assessment battery completion. These clinical aspects could have confounding effects on levels of temperamental and maladaptive personality traits together with their interrelationships within each clinical group. Ultimately, the lack of a precise assessment of the pharmacological treatments received by patients in each group did not allow us to control their potential impact on the psychological dimensions assessed in the present study. However, this limitation was expected, as the nature of the research hindered direct access to participants’ medical records, preventing accurate verification of this information. Therefore, future studies—particularly in clinical settings—should systematically assess the effects of pharmacological treatments for different forms of CP on temperamental and high‐order personality traits.

## 5. Conclusions

Despite these limitations, this is the first study that has provisionally highlighted core sensory processing sensitivity, temperamental and maladaptive personality traits, which capture different features of an altered emotional functioning, associated with NP mechanisms shared by a cluster of different CP syndromes. Furthermore, these provisional findings suggest focusing attention on dissociative/self‐related processes and dysfunctions in interpersonal functioning as possible key markers of a distinct subgroup of patients with comorbid CP conditions. Future longitudinal studies should clarify how SPS temperamental and maladaptive personality traits may interact with each other in sustaining different developmental pathways leading to specific CP conditions. Ultimately, this study supports a systematic assessment of maladaptive personality traits in CP conditions, especially to identify the best psychotherapeutic approach that could be added to well‐supported pharmacological treatments developed for each CP condition investigated in this study. According to the current findings, standard cognitive‐behavioral treatment or acceptance/mindfulness‐based programs might be considered the first‐line interventions for chronic headache, vulvodynia, and fibromyalgia [85, 86]. Whereas long‐term psychodynamic therapies (e.g., dynamic interpersonal therapy) or other evidence‐based treatments (e.g., dialectical behavior therapy), specifically developed to target dissociation and interpersonal difficulties [87, 88], may be more beneficial for patients with comorbid CP conditions.

## Ethics Statement

Ethical clearance for the project was obtained from the ethical committee of the Department of Dynamic and Clinical Psychology and Health Studies at Sapienza University of Rome, Italy, on November 25, 2022 [Protocol number 0001979 UOR: SI000092—Classified VII/15.

## Consent

Participants were aged 18 years or older. Accordingly, all participants personally provided informed consent prior to participation in the study.

## Conflicts of Interest

The authors declare no conflicts of interest.

## Author Contributions

Marco Cavicchioli: conceptualization, data curation, formal analysis, methodology, writing–original draft, and writing–review and editing.

Filippo Maria Nimbi: conceptualization, data curation, writing–original draft, and writing–review and editing.

Sara Bottiroli: writing–review and editing.

Daniele Guglielmi: data curation and writing–original draft.

Lorys Castelli: writing–review and editing.

Federica Galli: conceptualization, data curation, writing–original draft, and writing–review and editing.

## Funding

The current manuscript was related to the following project: “Relieving Chronic Pain: Psychosomatic Mechanisms and Psychological Interventions in Fibromyalgia and Chronic Headache (Fibromig Study)”—Code: 20229NZEKP; CUP Master: B53D23014410006; CUP Code B53D23014410006; Founded by the European Union (Next GenerationEU).

## Supporting Information

Additional supporting information can be found online in the Supporting Information section.

Supporting information reported the edge weights accuracy at 95% confidence intervals (CIs) computed by sampling the data 2000 times (with replacement), thereby generating a distribution of edge weights for each clinical group (i.e., Figures 3s–6s). This was estimated in order to test the significance of associations among edges of the network. The difference tests among centrality values were also estimated using the nonparametric bootstrapped difference test (i.e., Figures 1s–6s). This was evaluated in order to estimate the most representative temperamental and personality dimensions of each clinical and nonclinical group.

Figure 2s. Difference tests among centrality indexes in healthy controls.

Figure 3s. Difference tests among centrality in patients with chronic headache.

Figure 4s. Difference tests among centrality in patients with fibromyalgia.

Figure 5s. Difference tests among centrality in patients with vulvodynia.

Figure 6s. Difference tests among centrality in patients with comorbid chronic pain conditions.

Table 2s. Associations among temperamental and personological dimensions in the CH group estimated by the EBICglasso algorithm.

Table 3s. Associations among temperamental and personological dimensions in the VU group estimated by the EBICglasso algorithm.

Table 4s. Associations among temperamental and personological dimensions in the FM group estimated by the EBICglasso algorithm.

Table 5s. Associations among temperamental and personological dimensions in the COMPLEX group estimated by the EBICglasso algorithm.

## Supporting information


**Supporting Information 1** Figure 1s. Difference tests among centrality indexes in chronic pain conditions.

**Supporting Information 2** Table 1s. Associations among temperamental and personological dimensions in the CP group estimated by the EBICglasso algorithm.

## Data Availability

The data that support the findings of this study are available on request from the corresponding author (M.C.). The data are not publicly available due to privacy or ethical restrictions.
