# Correction to “Sensory Processing Sensitivity and Maladaptive Personality Traits in Chronic Pain Conditions: A Network Analysis Perspective”

**DOI:** 10.1155/prm/9838573

**Published:** 2026-07-29

M. Cavicchioli, F. Nimbi, S. Bottiroli, D. Guglielmi Castelli, L. Castelli, and F. Galli, “Sensory Processing Sensitivity and Maladaptive Personality Traits in Chronic Pain Conditions: A Network Analysis Perspective,” *Pain Research and Management* 2026 (2026), 8005108, https://doi.org/10.1155/prm/8005108.

In author list and related affiliations, the second author’s name Filippo Maria Nimbi was partially reported as “Filippo Nimbi.”

Furthermore, the reported affiliation “Department of Dynamic and Clinical Psychology, and Health Studies, Faculty of Medicine and Psychology, SAPIENZA University of Rome, Rome, Italy” is not corrected and should be substituted with “Department of Theoretical and Applied Sciences (DiSTA), eCampus University, Novedrate, Italy.”

We apologize for these errors.

