# Supplementary material for: Sensory Processing Sensitivity and Maladaptive Personality Traits in Chronic Pain Conditions: A Network Analysis Perspective
Source: Pain Res Manag. 2026 Mar 28;2026:8005108. doi: 10.1155/prm/8005108 (PMC13140176; doi:10.1155/prm/8005108)
Supplement: Supplementary file 1 — Supporting Information 1 Figure 1s. Difference tests among centrality indexes in chronic pain conditions. [file PRM-2026-8005108-s001.pptx]

## Slide 1
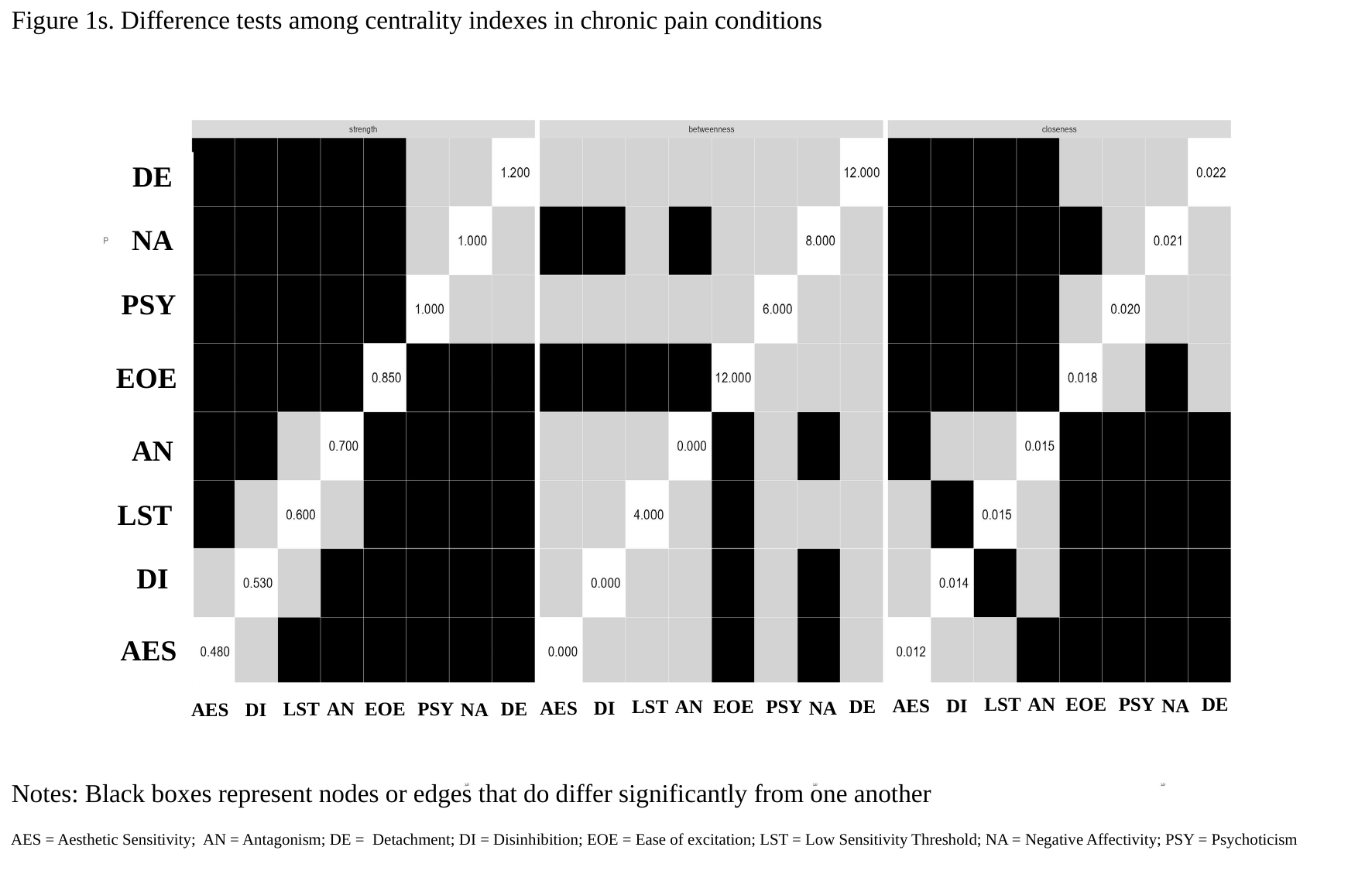

Figure 1s. Difference tests among centrality indexes in chronic pain conditions
DE
NA
PSY
EOE
AN
LST
DI
AES
LST
AN
EOE
PSY
DE
AES
DI
NA
LST
AN
EOE
PSY
DE
AES
DI
NA
LST
AN
EOE
PSY
DE
AES
DI
NA
Notes: Black boxes represent nodes or edges that do differ significantly from one another
AES = Aesthetic Sensitivity; AN = Antagonism; DE = Detachment; DI = Disinhibition; EOE = Ease of excitation; LST = Low Sensitivity Threshold; NA = Negative Affectivity; PSY = Psychoticism

## Slide 2
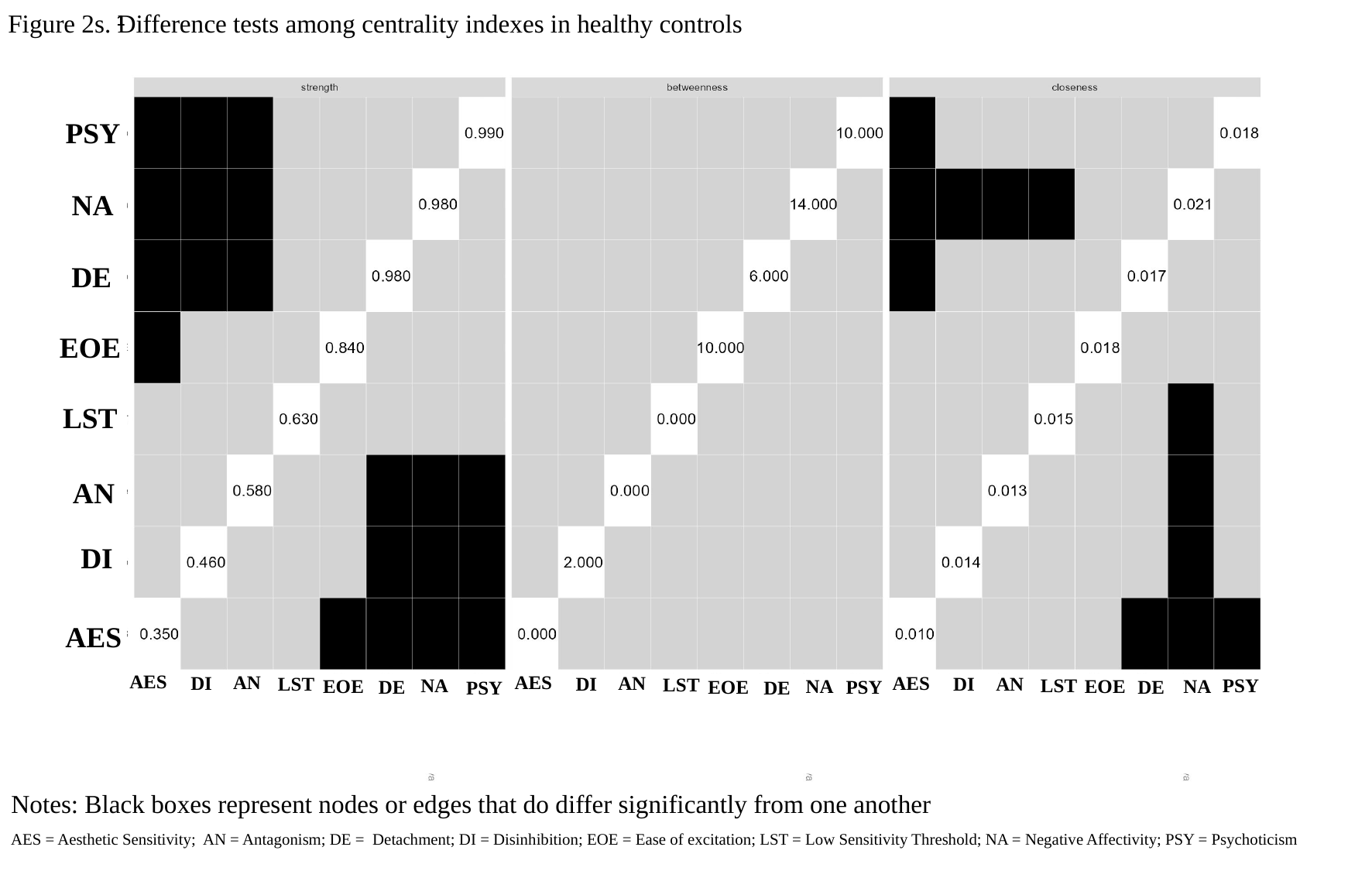

Figure 2s. Difference tests among centrality indexes in healthy controls
1
<
PSY
NA
DE
EOE
LST
AN
DI
AES
AES
AN
AES
DI
AES
AN
AN
DI
DI
LST
LST
PSY
LST
NA
NA
EOE
NA
EOE
DE
DE
EOE
PSY
DE
PSY
Notes: Black boxes represent nodes or edges that do differ significantly from one another
AES = Aesthetic Sensitivity; AN = Antagonism; DE = Detachment; DI = Disinhibition; EOE = Ease of excitation; LST = Low Sensitivity Threshold; NA = Negative Affectivity; PSY = Psychoticism

## Slide 3
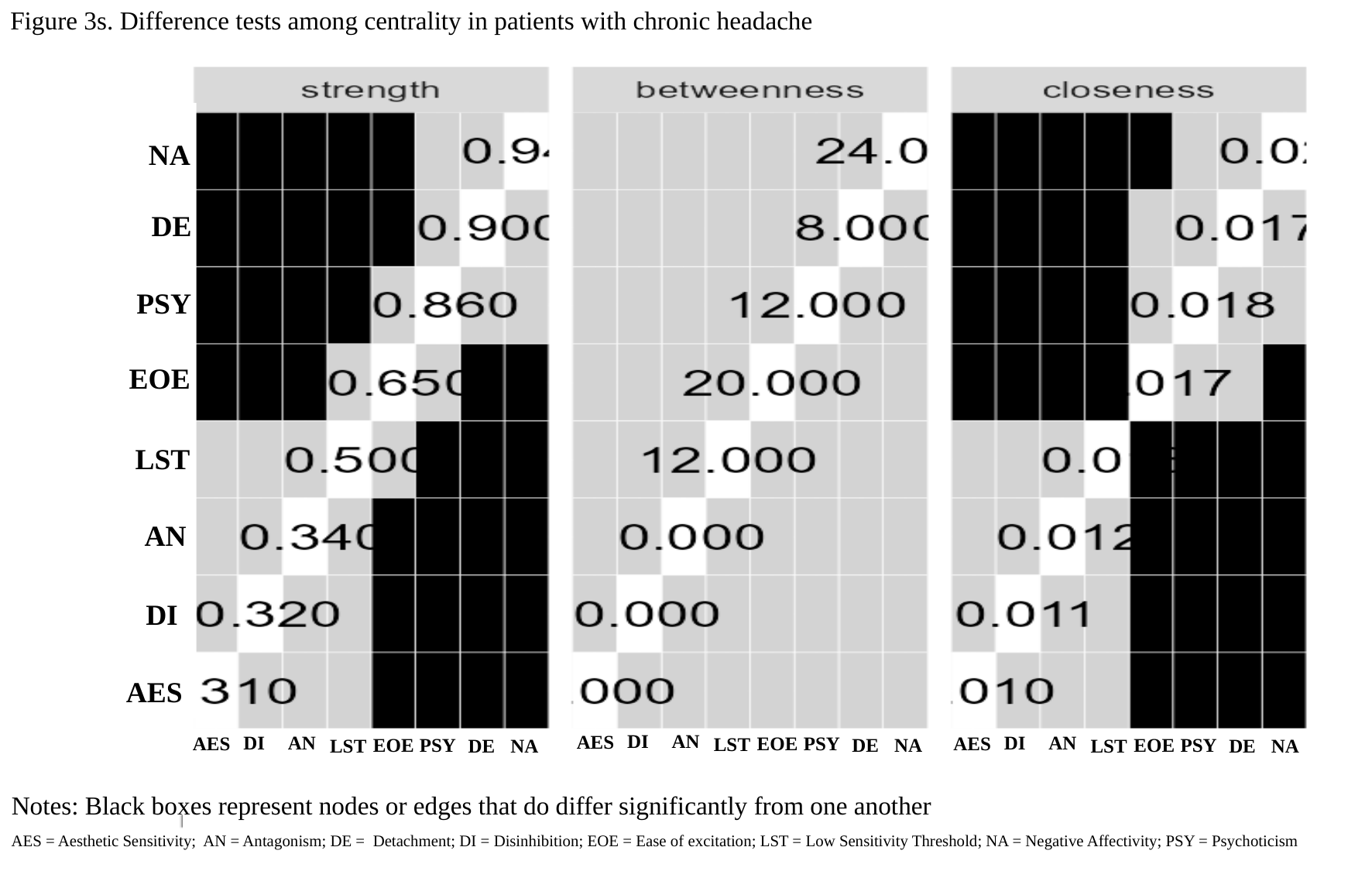

Figure 3s. Difference tests among centrality in patients with chronic headache
NA
DE
PSY
EOE
LST
AN
DI
AES
DI
AN
AES
DI
AN
DI
AN
AES
EOE
PSY
AES
LST
EOE
PSY
DE
NA
EOE
PSY
LST
LST
DE
NA
DE
NA
Notes: Black boxes represent nodes or edges that do differ significantly from one another
AES = Aesthetic Sensitivity; AN = Antagonism; DE = Detachment; DI = Disinhibition; EOE = Ease of excitation; LST = Low Sensitivity Threshold; NA = Negative Affectivity; PSY = Psychoticism

## Slide 4
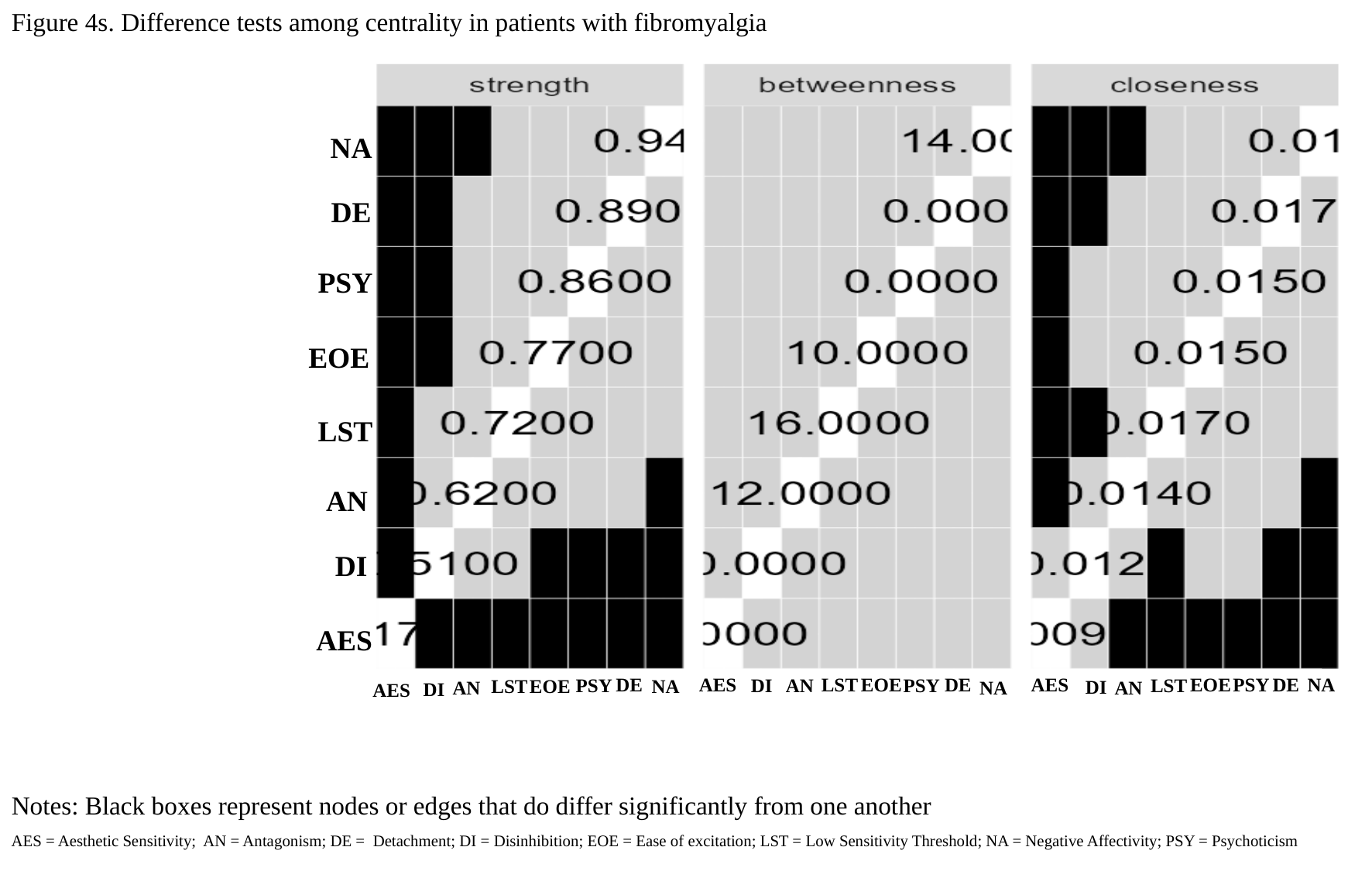

Figure 4s. Difference tests among centrality in patients with fibromyalgia
NA
DE
PSY
EOE
LST
AN
DI
AES
AES
NA
EOE
AES
DE
EOE
DE
PSY
LST
AES
DE
DI
LST
PSY
AN
PSY
NA
EOE
LST
DI
NA
AN
AN
DI
AES
Notes: Black boxes represent nodes or edges that do differ significantly from one another
AES = Aesthetic Sensitivity; AN = Antagonism; DE = Detachment; DI = Disinhibition; EOE = Ease of excitation; LST = Low Sensitivity Threshold; NA = Negative Affectivity; PSY = Psychoticism

## Slide 5
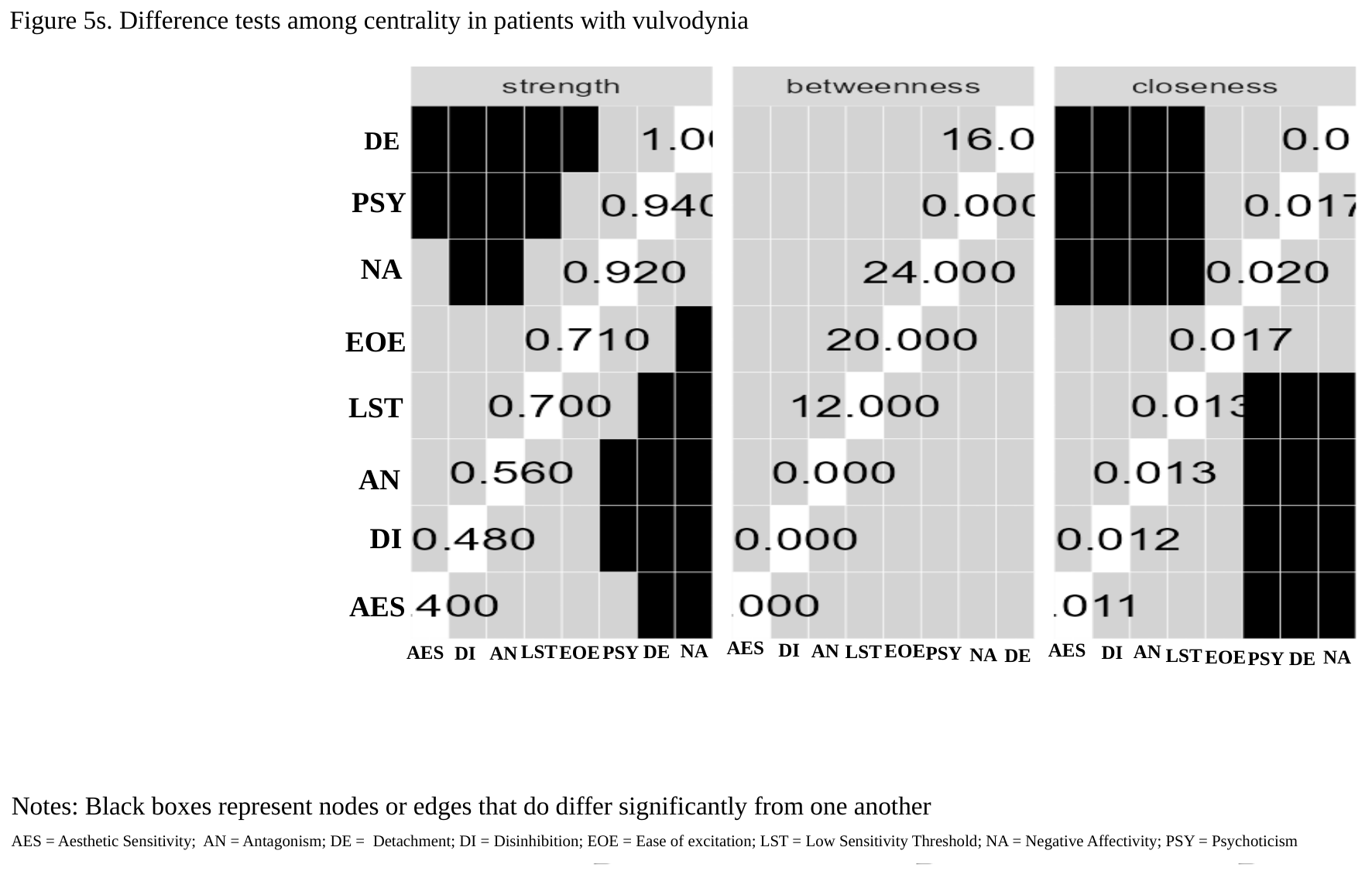

Figure 5s. Difference tests among centrality in patients with vulvodynia
DE
PSY
NA
EOE
LST
AN
DI
AES
AES
DI
AES
NA
AN
EOE
LST
LST
DE
AN
PSY
DI
EOE
AES
DI
AN
PSY
NA
DE
LST
EOE
NA
DE
PSY
Notes: Black boxes represent nodes or edges that do differ significantly from one another
AES = Aesthetic Sensitivity; AN = Antagonism; DE = Detachment; DI = Disinhibition; EOE = Ease of excitation; LST = Low Sensitivity Threshold; NA = Negative Affectivity; PSY = Psychoticism

## Slide 6
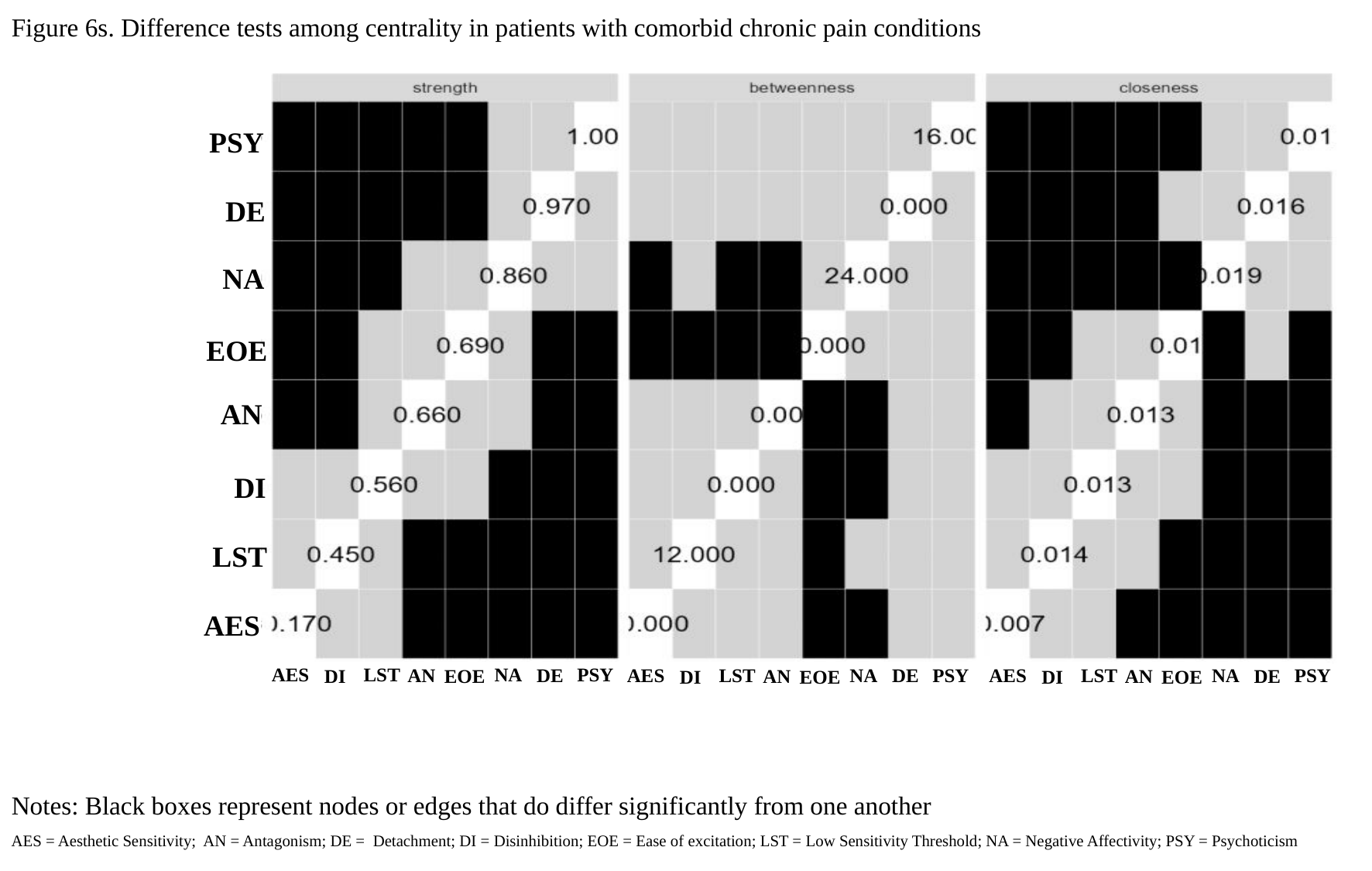

Figure 6s. Difference tests among centrality in patients with comorbid chronic pain conditions
PSY
DE
NA
EOE
AN
DI
LST
AES
LST
PSY
NA
AES
DE
LST
PSY
NA
AES
LST
AN
PSY
NA
DE
AES
DE
DI
AN
AN
EOE
DI
DI
EOE
EOE
Notes: Black boxes represent nodes or edges that do differ significantly from one another
AES = Aesthetic Sensitivity; AN = Antagonism; DE = Detachment; DI = Disinhibition; EOE = Ease of excitation; LST = Low Sensitivity Threshold; NA = Negative Affectivity; PSY = Psychoticism
