# Supplementary material for: Sensory Processing Sensitivity and Maladaptive Personality Traits in Chronic Pain Conditions: A Network Analysis Perspective
Source: Pain Res Manag. 2026 Mar 28;2026:8005108. doi: 10.1155/prm/8005108 (PMC13140176; doi:10.1155/prm/8005108)
Supplement: Supplementary file 2 — Supporting Information 2 Table 1s. Associations among temperamental and personological dimensions in the CP group estimated by the EBICglasso algorithm. [file PRM-2026-8005108-s002.docx]

Table 1s. Associations among temperamental and personological dimensions in CP group estimated by EBICglasso algorithm

|  | CP group | | | | | | | | HC group | | | | | | | |
| --- | --- | --- | --- | --- | --- | --- | --- | --- | --- | --- | --- | --- | --- | --- | --- | --- |
|  | EOE | LST | AES | NA | DE | AN | DI | PSY | EOE | LST | AES | NA | DE | AN | DI | PSY |
| EOE | - |  |  |  |  |  |  |  | - |  |  |  |  |  |  |  |
| LST | .09 | - |  |  |  |  |  |  | .11 | - |  |  |  |  |  |  |
| AES | .20 | .34 | - |  |  |  |  |  | .11 | .40 | - |  |  |  |  |  |
| NA | .03 | .24 | .02 | - |  |  |  |  | .00 | .21 | .00 | - |  |  |  |  |
| DE | .00 | -.05 | .00 | .08 | - |  |  |  | -.08 | .00 | .00 | .00 | - |  |  |  |
| ANT | .00 | .00 | .00 | .05 | .16 | - |  |  | -.05 | .00 | .00 | .14 | .03 | - |  |  |
| DI | -.10 | .14 | .04 | .27 | .22 | .14 | - |  | .00 | .08 | .10 | .27 | .23 | .11 | - |  |
| PSY | .05 | .00 | .00 | .31 | .19 | .18 | .26 | - | .00 | .04 | .01 | .36 | .12 | .26 | .20 | - |

AES = Aesthetic Sensitivity; ANT = Antagonism; DE = Detachment; DI = Disinhibition; EOE = Ease of arousal LST = Low Sensory Threshold; NA = Negative Affectivity; PSY = Psychoticism

Table 2s. Associations among temperamental and personological dimensions in CH group estimated by EBICglasso algorithm

|  | EOE | LST | AES | NA | DE | AN | DI | PSY |
| --- | --- | --- | --- | --- | --- | --- | --- | --- |
| EOE | - |  |  |  |  |  |  |  |
| LST | .08 | - |  |  |  |  |  |  |
| AES | .24 | .26 | - |  |  |  |  |  |
| NA | .00 | .21 | .01 | - |  |  |  |  |
| DE | .00 | .00 | .00 | .00 | - |  |  |  |
| ANT | .00 | .00 | .00 | .09 | .00 | - |  |  |
| DI | .00 | .10 | .00 | .33 | .19 | .05 | - |  |
| PSY | .00 | .00 | .00 | .30 | .15 | .18 | .23 | - |

AES = Aesthetic Sensitivity; ANT = Antagonism; DE = Detachment; DI = Disinhibition; EOE = Ease of arousal LST = Low Sensory Threshold; NA = Negative Affectivity; PSY = Psychoticism

Table 3s. Associations among temperamental and personological dimensions in VU group estimated by EBICglasso algorithm

|  | EOE | LST | AES | NA | DE | AN | DI | PSY |
| --- | --- | --- | --- | --- | --- | --- | --- | --- |
| EOE | - |  |  |  |  |  |  |  |
| LST | .04 | - |  |  |  |  |  |  |
| AES | .34 | .26 | - |  |  |  |  |  |
| NA | .00 | .22 | .06 | - |  |  |  |  |
| DE | .00 | .00 | .00 | .08 | - |  |  |  |
| ANT | .00 | .03 | .00 | .03 | .11 | - |  |  |
| DI | .00 | .10 | .00 | .29 | .19 | .17 | - |  |
| PSY | .02 | .06 | .04 | .23 | .17 | .14 | .28 | - |

AES = Aesthetic Sensitivity; ANT = Antagonism; DE = Detachment; DI = Disinhibition; EOE = Ease of arousal LST = Low Sensory Threshold; NA = Negative Affectivity; PSY = Psychoticism

Table 4s. Associations among temperamental and personological dimensions in FM group estimated by EBICglasso algorithm

|  | EOE | LST | AES | NA | DE | AN | DI | PSY |
| --- | --- | --- | --- | --- | --- | --- | --- | --- |
| EOE | - |  |  |  |  |  |  |  |
| LST | .01 | - |  |  |  |  |  |  |
| AES | .16 | .30 | - |  |  |  |  |  |
| NA | .00 | .19 | .08 | - |  |  |  |  |
| DE | .00 | .00 | .00 | .22 | - |  |  |  |
| ANT | .00 | .00 | .00 | .05 | .25 | - |  |  |
| DI | .00 | .14 | .00 | .23 | .14 | .12 | - |  |
| PSY | .00 | .07 | .08 | .18 | .17 | .09 | .26 | - |

AES = Aesthetic Sensitivity; ANT = Antagonism; DE = Detachment; DI = Disinhibition; EOE = Ease of arousal LST = Low Sensory Threshold; NA = Negative Affectivity; PSY = Psychoticism

Table 5s. Associations among temperamental and personological dimensions in COMPLEX group estimated by EBICglasso algorithm

|  | EOE | LST | AES | NA | DE | AN | DI | PSY |
| --- | --- | --- | --- | --- | --- | --- | --- | --- |
| EOE | - |  |  |  |  |  |  |  |
| LST | .01 | - |  |  |  |  |  |  |
| AES | .16 | .30 | - |  |  |  |  |  |
| NA | .00 | .19 | .08 | - |  |  |  |  |
| DE | .00 | .00 | .00 | .22 | - |  |  |  |
| ANT | .00 | .00 | .00 | .05 | .25 | - |  |  |
| DI | .00 | .14 | .00 | .23 | .14 | .12 | - |  |
| PSY | .00 | .07 | .08 | .18 | .17 | .09 | .26 | - |

AES = Aesthetic Sensitivity; ANT = Antagonism; DE = Detachment; DI = Disinhibition; EOE = Ease of arousal LST = Low Sensory Threshold; NA = Negative Affectivity; PSY = Psychoticism
